# A Fast and Easy-To-Go Method for the Preparation of Au Nanocluster and Its Application for Fe(III) Cation Sensing

**DOI:** 10.3389/fchem.2021.794725

**Published:** 2021-12-07

**Authors:** Peibin Zhu, Wen Chen, Liang Liu

**Affiliations:** ^1^ School of Ocean Information Engineering, Jimei University, Xiamen, China; ^2^ School of Materials Science & Engineering, Jiangsu University, Zhenjiang, China

**Keywords:** AuNCs, optical sensing, emission quenching, Fe(III) detection, LMNCT

## Abstract

In this article, we reported the synthesis and characterization of gold nanoclusters (AuNCs) with a diameter of ∼2 nm. A simple method of microwave-assisted reaction was applied here, with L-cysteine as both reducing agent and stabilizer. The resulting AuNCs were analyzed by means of TEM, XPS, DLS, and IR. Their photophysical performance was then analyzed in detail, including UV-vis absorption, emission, quantum yield, and lifetime. Efficient red emission was observed from these AuNCs, originating from ligand-to-metal nanoparticle core charge transfer (LMNCT). This red emission was found quenchable by Fe(III) cations. The corresponding quenching curve and sensing performance were discussed. An effective working region of 0–80 μM with an LOD of 3.9 μM was finally observed. Their quenching mechanism was revealed as Fe(III) energy competing for the LMNCT process. The novelty and advancement of this work is the simple synthesis and impressive sensing performance, including wide working region, good linearity, and selectivity.

## Introduction

As a class of novel nanomaterials, luminescent metal nanoclusters have drawn much research attention in fields of optical sensing, imaging, biolabeling, and therapy (Nandi et al., 2018; Zhang et al., 2018). Since the size of these nanoclusters is comparable to the Fermi wavelength of electrons, molecular-like behavior is usually observed from these nanoclusters such as separated electronic states and size-dependent electronic transitions, which endows them with different optical features from those of nanoparticles. Among these metal nanoclusters, gold nanoclusters (AuNCs) have shown promising potential in nanotechnology and biotechnology, especially optical sensing and imaging (Yau et al., 2013). There have been studies focusing on the synthesis and application of AuNCs, including their luminescence performance and sensing mechanism (Huang et al., 2007; Wu and Jin, 2010). For example, Wu and coworkers have reported AuNCs encapsulated by the thiolate ligand and their enhanced luminescence (Wu and Jin, 2010). Regardless of the promising result, this synthetic strategy needs long reaction time, high reaction temperature, and multiple reagents. Ying and coworkers have reported a “green” method for the synthesis of AuNCs using bovine serum albumin (BSA) as both a reducing agent and stabilizer, resulting in highly luminescent AuNCs (Xie et al., 2009). Similarly, Zhang and coworkers have synthesized luminescent AuNCs with the help of enzymes (horseradish peroxidase) (Wen et al., 2011). The resulting luminescent AuNCs have been explored as a biosensor to determine H_2_O_2_. Up to now, the development of easy-to-go and “green” synthetic method for AuNCs with good stability, environmental friendliness, high biocompatibility, and strong emission is still a hot topic for nanomaterials.

It has been reported that microwave irradiation can be used as a heating source to accelerate chemical reactions (Gedye et al., 1986). Thereafter, many efforts have been devoted to microwave-assisted reactions. It has been reported that microwave-assisted reactions have shown advantages of uniform radiation, fast speed, limited energy consumption, and low cost (Beeri et al., 1986). These features make the microwave-assisted reaction an attractive method for the synthesis of nanomaterials as well, since it shortens reaction time and yields uniform products in terms of size and composition (Buhler and Feldmann, 2006). For example, Buhler and coworkers first synthesized luminescent nanocrystals *via* a microwave-assisted reaction (Buhler and Feldmann, 2006). Yu and coworkers reported a microwave-assisted reaction for the synthesis of Fe_2_O_3_ nanorings without any templates or surfactants (Hu et al., 2007). Gold nanoparticles have been successfully prepared without any reducing agent *via* a microwave-assisted reaction, as reported by Yacaman (Vargas–Hernandez et al., 2010). Lately, AuNCs have been prepared *via* such microwave-assisted reaction as well. For example, Li and coworkers reported a one-step method for the preparation of AuNCs and its application for Ag(I) sensing *via* fluorescence quenching (Yuan et al., 2012). Choi and coworkers reported BSA- and HAS-protected AuNCs with strong red emission (Yan et al., 2012). These reports spark further exploration of luminescent AuNCs and their application.

**SCHEME 1:**
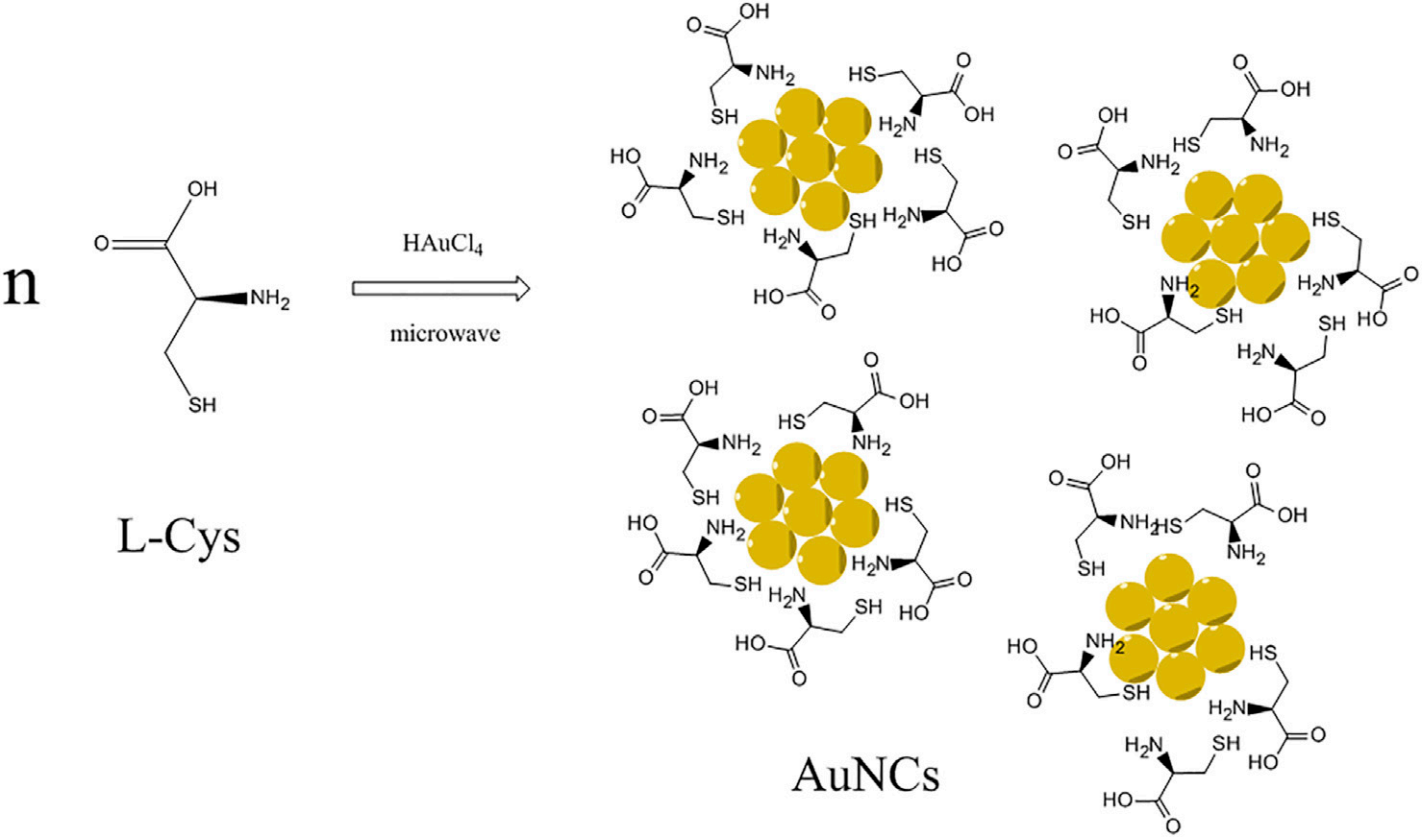
Synthetic route for AuNCs *via* microwave-assisted reaction using L-cysteine as both a reducing agent and stabilizer.

In this study, AuNCs were synthesized *via* a microwave-assisted reaction using L-cysteine as both a reducing agent and stabilizer, as depicted in Scheme 1. The resulting AuNCs were characterized carefully by means of TEM, XPS, DLS, and IR. Their photophysical performance was then analyzed in detail, including UV-vis absorption, emission, quantum yield, and lifetime. Efficient red emission was observed from these AuNCs and then found quenchable by Fe(III) cation. The corresponding quenching curve and sensing performance were discussed.

## Experimental Section

### Reagents and Equipment

All chemicals and reagents used in this work were AR grade 1 unless noticed and purchased from Sigma-Aldrich Chemical Corporation, including HAuCl_4_, L-cysteine (L-Cys) and anhydrous FeCl_3_. Other inorganic chemicals were purchased from Tianjin Chemical Corporation. Solvent water was purified and obtained from Millipore. All glassware was first treated with aqua regia at 40°C and then washed with purified water. XPS and DLS experiments were carried out by a PHI-5000 Versaprobe III X-ray photon–electron spectrophotometer (ULVCA-PHI, United States) and a Malvern Zetasizer Nano ZS90 laser particle size and zeta potential analyzer (Trek, Taiwan). IR spectra were recorded using a Bruker Vertex 70 FTIR spectrometer. The TEM image was obtained with a JEM-2010 transmission electron microscope (JEOL, Japan). UV-vis absorption spectra were obtained using a Shimadzu UV-3101PC spectrophotometer and a Biotek ELx800 automatic enzyme-linked immunosorbent assay. Emission spectra were recorded using a Hitachi F-7000 fluorescence spectrophotometer (Xe lamp). Emission quantum yield was determined by using this Hitachi F-7000 fluorescence spectrophotometer with the help of an integrating sphere. The reference was quinine sulfate (20 μM) in diluted H_2_SO_4_ (0.1 M). The emission lifetime was measured with a TEKTRONIX TDS-3052 oscilloscope (excited by an optical parametric oscillator).

### Synthesis of AuNCs

AuNCs were synthesized following a literature procedure with minor revisions (Yan et al., 2012). For the synthesis, 10 ml of HAuCl_4_ aqueous solution (10 mM) was mixed with 2 ml of L-Cys aqueous solution (3 mM) and then treated with ultrasonic bath for 5 min. Then this mixture was placed in a microwave reactor (in a representative run, the microwave energy was set as 5 W) for 120 s. The resulting solution was cooled naturally and obtained as AuNC aqueous solution. This crude product was kept in a refrigerator (4°C). Before further characterization and performance test, it was diluted with water to avoid sample self-aggregation or self-absorption.

### Measurement of AuNC Sensing Performance

For sensing performance measurement, the metal cations were first dissolved in pure water and then mixed with AuNC aqueous solutions. After a complete mixing, each sample was measured using the F-7000 fluorescence spectrophotometer. Sensing performance was based on steady emission spectra.

## Results and Discussion

### Optimization of AuNC Synthesis

**FIGURE 1:**
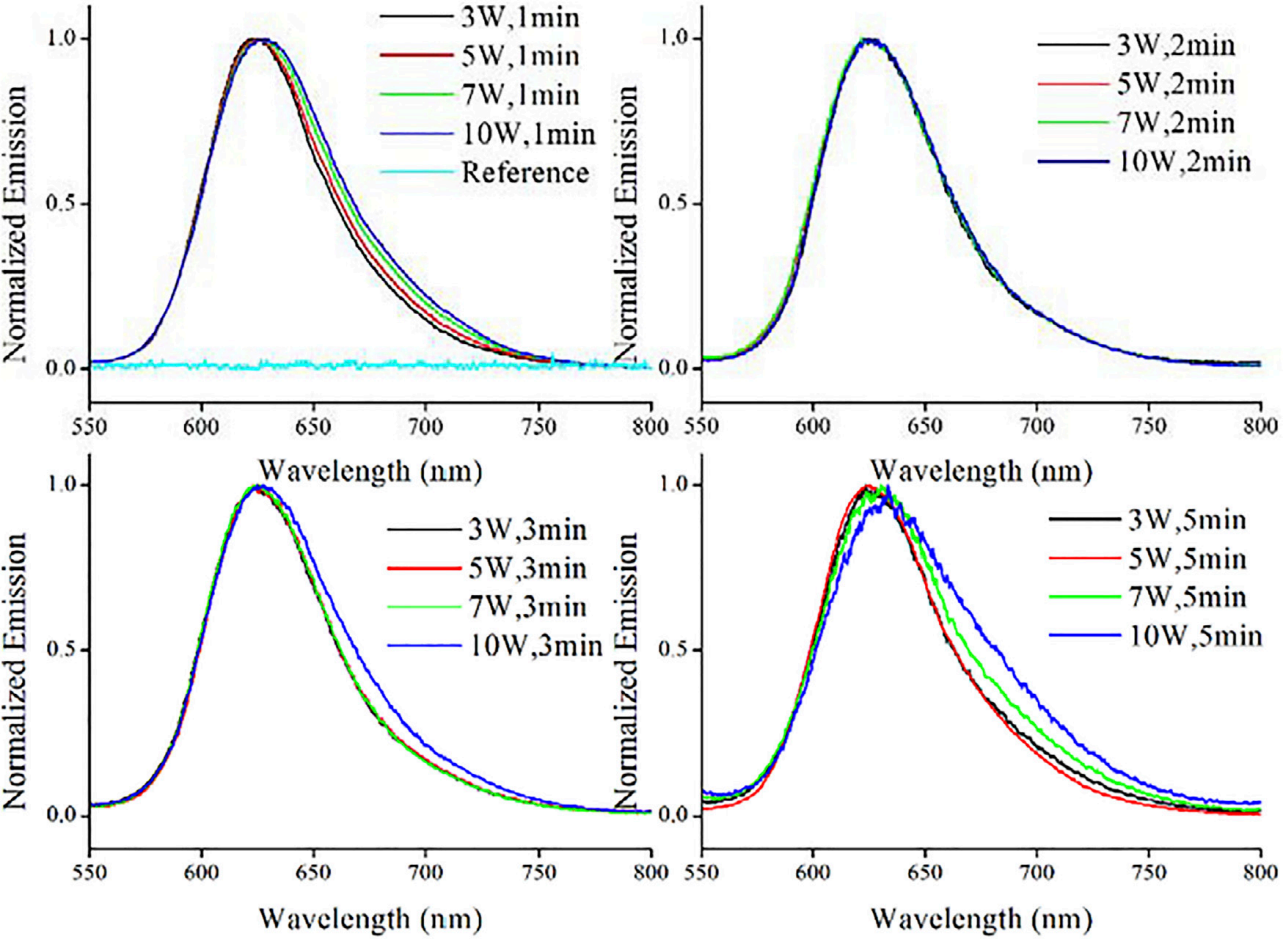
Emission spectra of AuNCs prepared under various conditions and a reference sample (pure L-Cys treated with microwave), diluted with pure water (v:v = 1:10).

The synthetic condition optimization of AuNCs was finished by varying microwave power and reaction time. Corresponding photophysical parameters of resulting AuNCs are recorded and compared in Table 1, including emission wavelength (λ_em_), FWHM (full width at half maximum) and emission quantum yield (Φ). The emission spectra of resulting AuNCs are shown in Figure 1. It is observed that upon various synthetic conditions of microwave radiation power and reaction time, these AuNC samples exhibit red emission ranging from 574 to 582 nm. A single emission band is observed for each AuNC sample, with no shoulder emission or vibronic progressions. For comparison, pure L-Cys was treated with microwave and then sent to record its emission spectrum (referred as reference sample). No valid emission signal is observed for this reference sample, as shown in Figure 1, indicating that the red emission comes from AuNCs, instead of L-Cys or its degrade products. Given the same reaction time, both λ_em_ and FWHM are affected by microwave radiation power. It appears that λ_em_ shows red shift tendency with increasing microwave radiation power; meanwhile, FWHM is slightly increased. On the other hand, if the microwave radiation power is fixed, then both λ_em_ and FWHM are independent of reaction time, showing very small variation upon increasing reaction time, as shown in Table 1. In other words, the emission feature of AuNCs is mainly dominated by microwave radiation power. As observed from Table 1, the optimal synthetic condition is found as a microwave radiation power of 5 W and reaction time of 2 min, showing the highest emission quantum yield of 17.6%. With microwave radiation power weaker than 5 W, no enough AuNCs are synthesized, leading to underdeveloped emission quantum yield. If microwave radiation power is higher than 5 W, the as-synthesized AuNCs may be decomposed by excess microwave radiation, compromising emission quantum yield as well. This explains why AuNCs prepared under a microwave radiation power of 10 W and reaction time of 5 min show the lowest emission quantum yield of only 2.5%. For later measurement, effort is focused on the optimal sample of AuNCs prepared under a microwave radiation power of 5 W and reaction time of 2 min.

**TABLE 1:** Photophysical parameters of AuNCs upon various microwave radiation powder and reaction time.

Power, time	λ_em_ (nm)	FWHM (nm)	Φ (%)
3 W, 1 min	624	62	12.1
5 W, 1 min	625	65	13.2
7 W, 1 min	627	68	10.5
10 W, 1 min	628	69	7.5
3 W, 2 min	626	63	15.3
5 W, 2 min	625	65	17.6
7 W, 2 min	526	66	11.3
10 W, 2 min	527	65	10.1
3 W, 3 min	624	63	14.7
5 W, 3 min	625	64	15.4
7 W, 3 min	626	65	8.3
10 W, 3 min	627	69	6.2
3 W, 5 min	625	63	11.0
5 W, 5 min	625	65	9.4
7 W, 5 min	631	70	5.8
10 W, 5 min	632	82	2.5
Reference sample	No emission	N/A	N/A

### Characterization of As-Synthesized AuNCs

#### XPS and TEM

**FIGURE 2:**
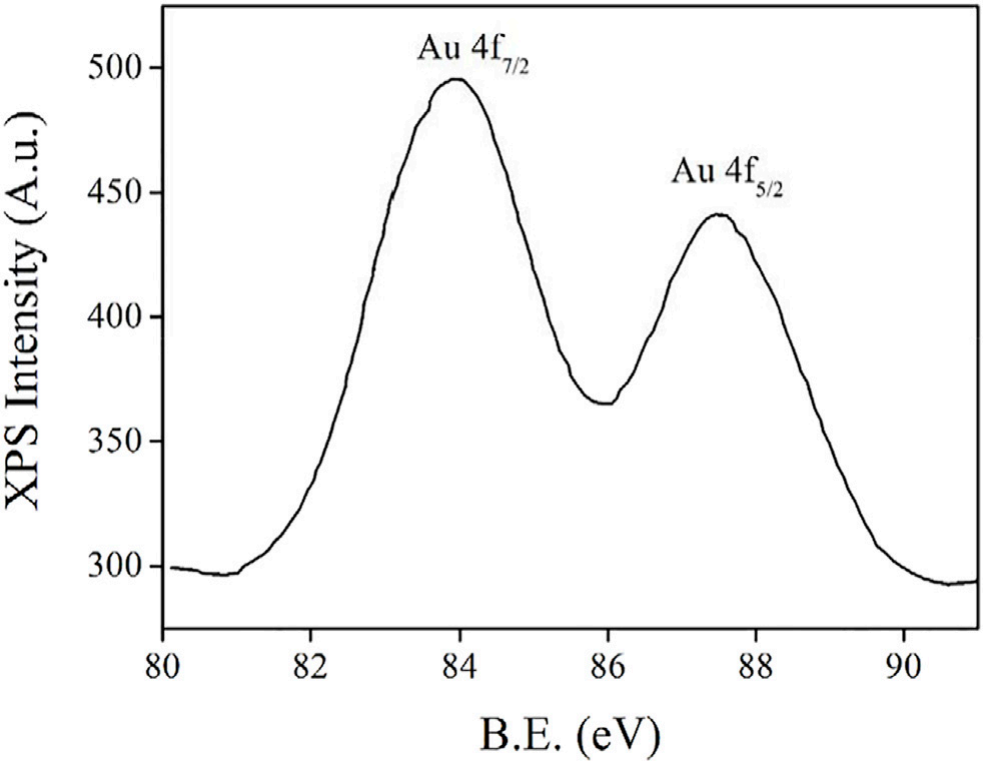
XPS spectrum of the AuNCs.

For a better understanding on the as-synthesized AuNCs, further characterization is performed as follows. First, their oxidation state is revealed using their XPS data shown in Figure 2. There are two XPS peaks at 84.0 and 87.5 eV, respectively, corresponding to Au 4f_7/2_ and Au 4f_5/2_ peaks (He et al., 2011). These two peaks match with metallic Au(0), indicating the successful synthesis of AuNCs. The ΔBE value between these two peaks is 3.5 eV, which tentatively confirms that elemental AuNCs have been trapped within the framework formed by L-Cys (Betzig, 2015). In addition, it has been reported that the XPS values of Au 4f_7/2_ and Au 4f_5/2_ peaks depend on the size of AuNCs since there are two structural components in AuNCs, inner and surface Au atoms (Tanaka et al., 2003). With the decreasing core size of AuNCs, the Au 4f_7/2_ peak for inner Au atoms is gradually increased from 84.0 to 84.3 eV, while the Au 4f_7/2_ peak for surface Au atoms (84.3–84.7 eV) is always higher than that for inner Au atoms. Since the recorded Au 4f_7/2_ value is as low as 84.0 eV, it is safe to say that these Au atoms are aggregated as nanoclusters successfully.

**FIGURE 3:**
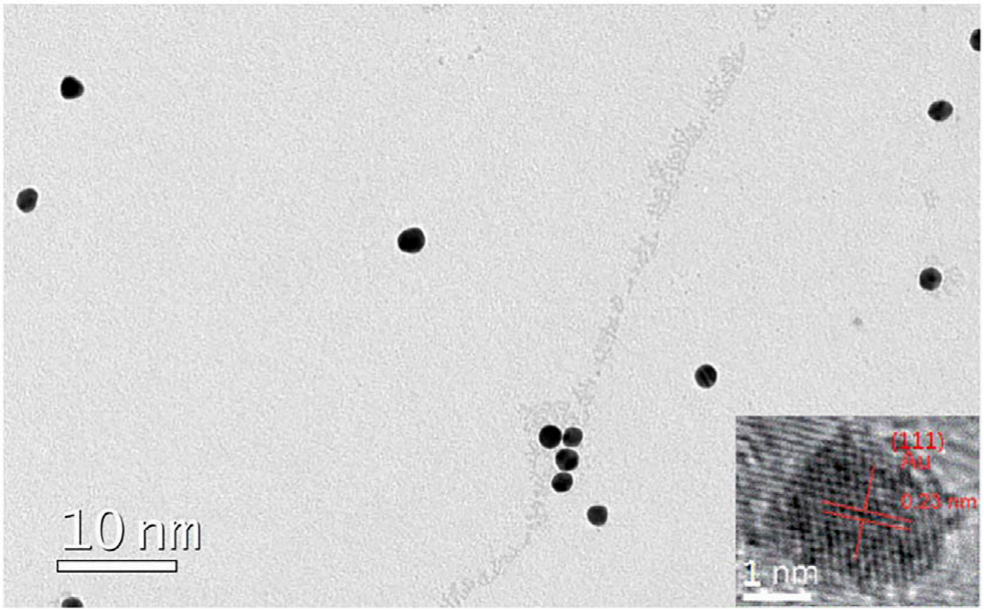
TEM image of the AuNCs.

A visual confirmation for the successful synthesis of AuNCs is provided in Figure 3. These nanoclusters are uniformly dispersed, showing an average diameter of 2 nm. A typical lattice fringe is measured as 0.23 nm, which agrees well with the lattice spacing of metallic Au (d spacing of 111 crystal plane) (Buffat et al., 1991). This observation is consistent with the aforementioned hypothesis that Au atoms are aggregated as nanoclusters.

#### DLS and IR Spectra

**FIGURE 4:**
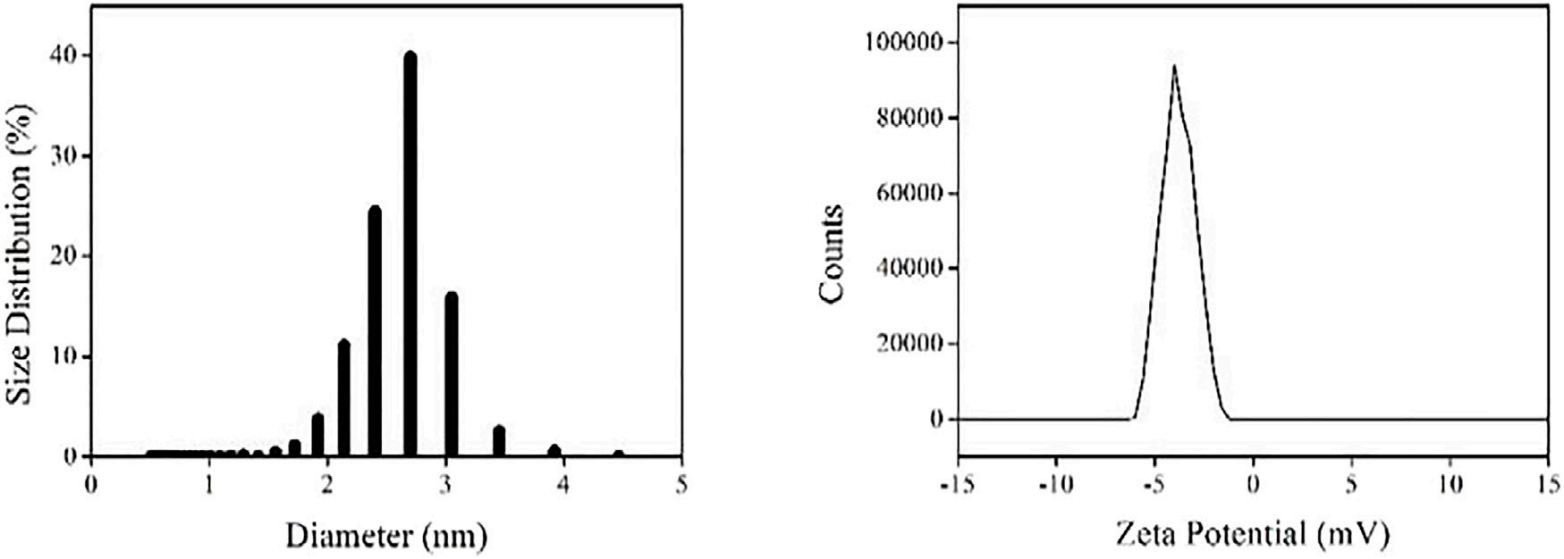
DLS (left) and Zeta potential (right) of the AuNCs dispersed in pure water (v:v = 1:10).

For a better understanding on the size distribution of these as-synthesized AuNCs, their dynamic light scattering (DLS) is determined. It is observed from Figure 4 that most AuNCs localize in a narrow size region of 2.1–3.1 nm. The dominant component of these AuNCs is those with size of 2.7 nm. This value is slightly higher than that observed from the TEM result (∼2 nm). This is because the L-Cys framework outside of AuNCs increases the particle size during DLS measurement. Nevertheless, this result still confirms the successful synthesis of AuNCs. The corresponding zeta potential is measured as −4.0 ± 0.5 mV, indicating a good stability of these AuNCs. This value is found lower than values given in the literature (∼−20 mV) of AuNCs prepared with strong reducing agents such as NaBH_4_ (Zhang et al., 2014; Helmbrecht et al., 2015).

**FIGURE 5:**
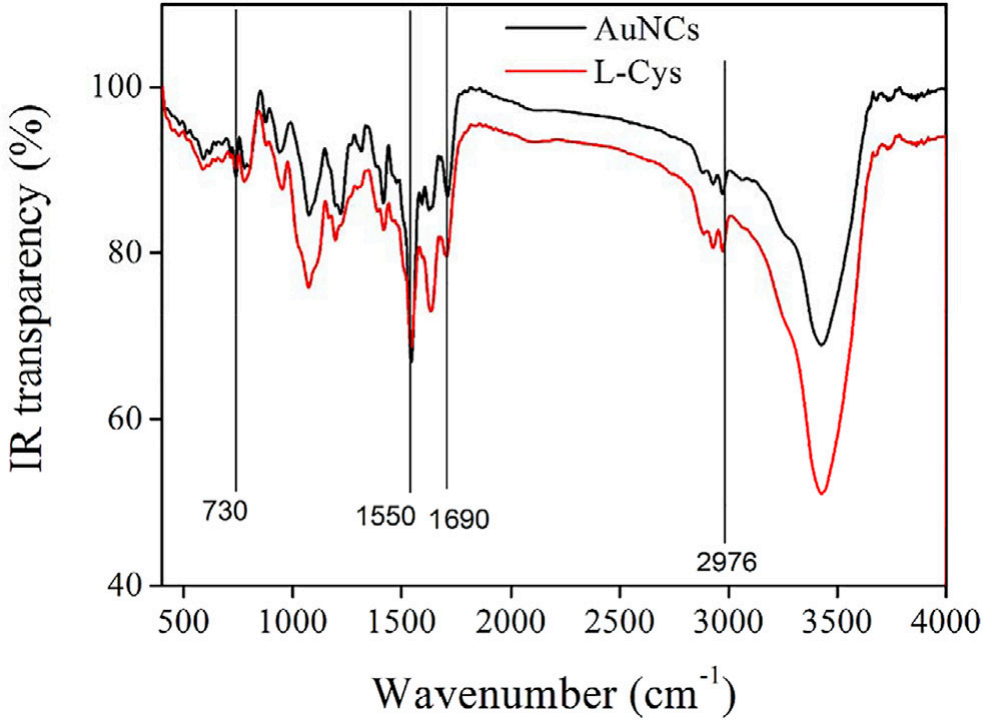
IR spectra of L-Cys and as-synthesized AuNCs.

IR spectra of L-Cys and as-synthesized AuNCs are recorded and shown as Figure 5. As for L-Cys, there is a characteristic IR band at 1690 cm^−1^ which is attributed to the amide I band of L-Cys. Another IR band centering at 1550 cm^−1^ is regarded as strong primary amine scissoring absorption. C-H vibration of L-Cys is responsible for the IR bands around 2976 cm^−1^. The weak IR absorption at ∼730 cm^−1^ is considered as the absorption of –NH_2_ wagging. These characteristic bands are consistent with the molecular structure of L-Cys. All these IR bands can be found from the IR spectrum of AuNCs, with a slim spectra shift, indicating that microwave heating exerts a small effect on the surface electric property of L-Cys during the synthesis of AuNCs. The existence of AuNCs affects the surface electric property of L-Cys. Considering the observation of L-Cys–derived IR peaks in these AuNCs, it is assumed that these AuNCs should be covered and protected by L-Cys but not the carbon shell, as depicted by Scheme 1. Such L-Cys shells are important for the stability of AuNCs, and it should not be removed. If so, these AuNCs aggregate together and lose their emission feature.

### Photophysical Performance of As-Synthesized AuNCs

#### UV-Vis Absorption and Excitation Spectra

**FIGURE 6:**
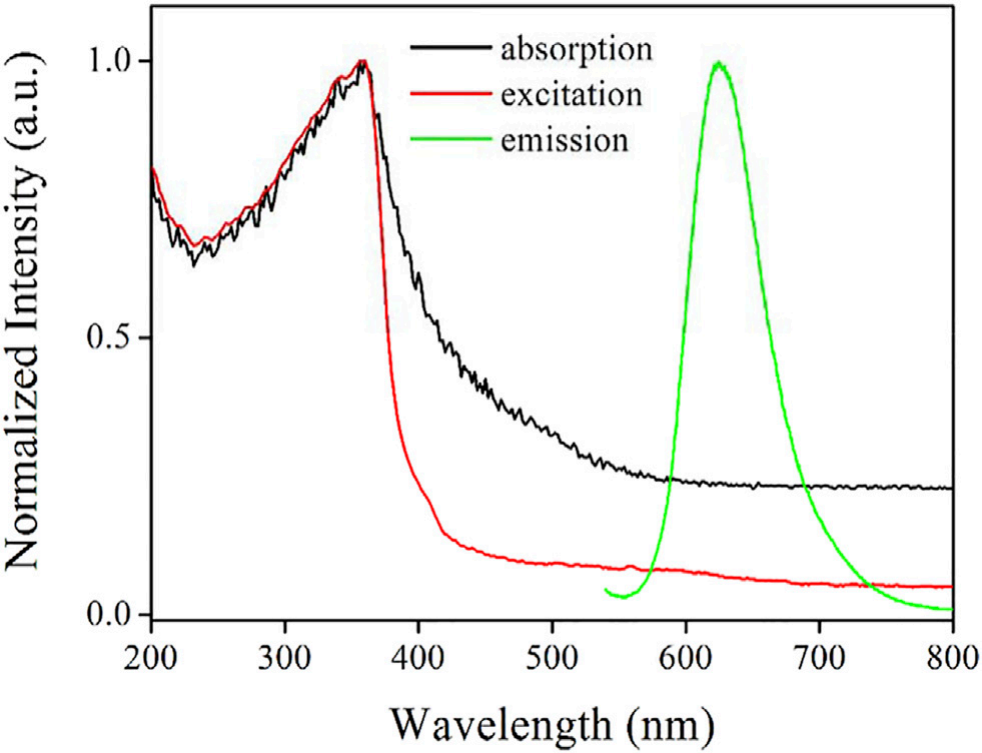
Absorption, excitation, and emission of the AuNCs diluted by pure water (v:v = 1:10).

It is observed from Figure 6 that the as-synthesized AuNCs exhibit obvious electronic absorption features, unlike the simple absorption from AuNCs synthesized under ambient conditions (Shang et al., 2012). A strong absorption band peaking at 360 nm is observed, extending to the visible region of ∼600 nm. The high background absorption noise (absorbance = ∼0.2) within 600–800 nm is attributed to the light scattering effect of AuNCs. It has been widely reported that Au nanoparticles generally exhibit an absorption band of plasmon resonance at ∼522 nm (Helmbrecht et al., 2015). No such absorption around 520 nm is observed for the as-synthesized AuNCs though, which further confirms the successful synthesis of AuNCs.

Correspondingly, the excitation spectrum of the as-synthesized AuNCs is nearly identical with its absorption band, peaking at 358 nm. With the elimination of scattering light, this excitation spectrum shows low background noise. This transition has been reported as intraband (sp to sp) transition within AuNCs and also used as a proof to conform the existence of AuNCs. A highly fluorescent transition shall be resulted by this intraband transition, along with the mixed interband (d to sp) transition (Shang et al., 2012). This statement will be confirmed later.

#### Emission Features

It is observed from Figure 6 that the as-synthesized AuNCs exhibit a Guassian-like emission band upon excitation wavelength of 360 nm, with an emission peak of 625 nm. Their FWHM and emission quantum yield have been measured as 65 nm and 17.6%, respectively, as listed in Table 1. This emission quantum yield is found higher than values of similar AuNCs given in the literature (lower than 10%) (Zhang et al., 2014). A broad emission contribution is observed at a longer wavelength (>650 nm), leading to the high FWHM value (65 nm), which can be attributed to the heterogeneous size of AuNCs. On the other hand, this broad emission band indicates that its emission indeed comes from the fluorescent AuNCs trapped in the framework of L-Cys, which are generated by the *in situ* reduction of Au(III) to Au(0), as reported by Xie and coworkers (Xie et al., 2007). The Stokes shift between emission peak (625 nm) and excitation peak (360 nm) is as large as 265 nm and comparable to the values given in the literature (∼270 nm) (Xie et al., 2007; Zhang et al., 2014; Helmbrecht et al., 2015). This large Stokes shift makes the interference from excitation light negligible, which favors its further optical application.

**FIGURE 7:**
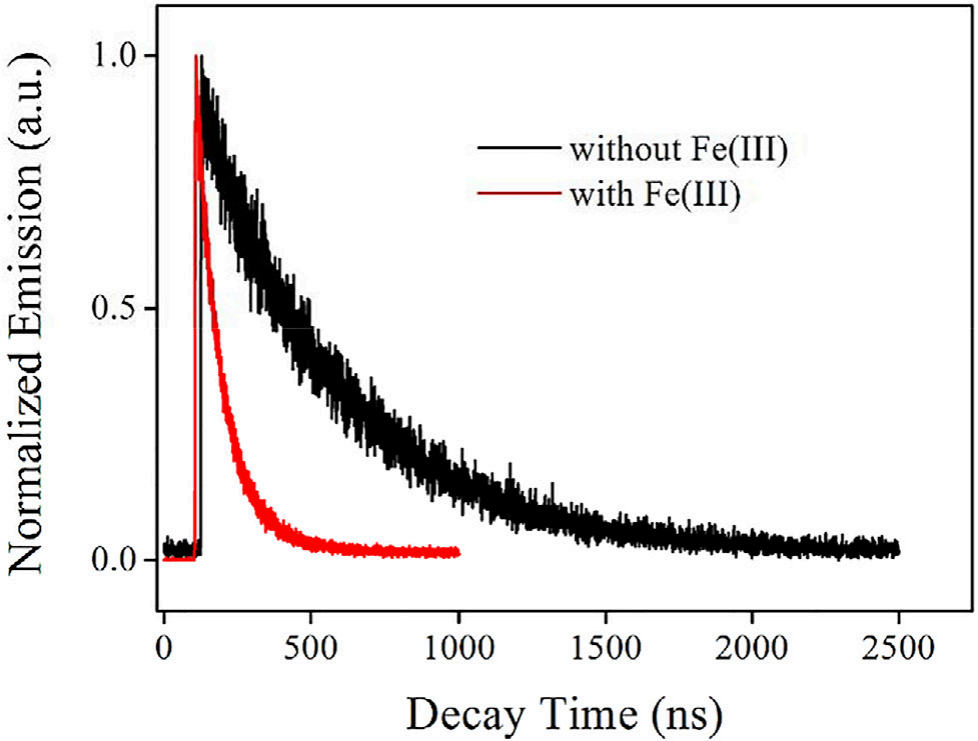
Emission decay dynamics curve of the AuNCs diluted by pure water (v:v = 1:10), without and with Fe(III) (100 μM).

For a better understanding of the emissive state of the as-synthesized AuNCs, their emission decay dynamics curve is recorded and shown as Figure 7. It is observed from Table 2 that the emission follows biexponential decay pattern, with an average decay lifetime (τ) of 466.99 ns. There are two emissive decay centers in AuNCs, a fast-decay one and a slow-decay one. This observation is consistent with the emission mechanism of ligand to the metal nanoparticle core charge transfer (LMNCT) (Chang et al., 2013). This fast-decay component is tentatively assigned as the direct transition of AuNCs (sp to sp or d to sp), while the slow-decay component is regarded as the transition of LMNCT. With the emission quantum yield on hand (Φ = 17.6%), radiative and non-radiative probabilities (K_r_ and K_nr_) can be calculated with Formula 1 and Formula 2. Here, Φ means emission quantum yield and τ means average emission decay lifetime. The K_r_ value is as high as 3.77 × 10^5^ s^−1^, indicating a spin-allowed transition, which matches the proposed LMNCT mechanism:

$$\Phi =\frac{k_{r}}{k_{r}+k_{nr}}$$ (1)

$$\frac{1}{\tau }=k_{r}+k_{nr}$$ (2)

**TABLE 2:** Fitting parameters for the emission decay dynamic curves of AuNCs.

AuNC	τ_1_ (ns)	A_1_	τ_2_ (ns)	A_2_	τ (ns)	K_r_ (s^−1^)	K_nr_ (s^−1^)
No Fe^3+^	477.67	1.14	65.89	0.22	466.99	3.77 × 10^5^	1.76 × 10^6^
With Fe^3+^	149.34	0.35	78.76	3.06	91.34	3.83 × 10^5^	1.06 × 10^7^

The number of Au atoms in these as-synthesized AuNCs is tentatively determined by the jellium model, which is described by Formula 3. Here, ν means the frequency of emission/excitation light, E_f_ denotes Fermi energy, N means the number of atoms in nanocluster, R is the diameter of this nanocluster, and r_s_ stands for the Wigner–Seitz radius, respectively. With the excitation and emission spectra on hand, the number of Au atoms in these as-synthesized AuNCs is determined as 28, which matches their diameter of ∼2 nm as shown in Figure 3.

$$\text{hν}\approx \frac{E_{f}}{\sqrt[{3}]{N}}=\frac{\left(E_{f}r_{s}\right)}{R}$$ (3)

The sensing performance of as-synthesized AuNCs toward Fe(III).

#### Emission Spectra

**FIGURE 8:**
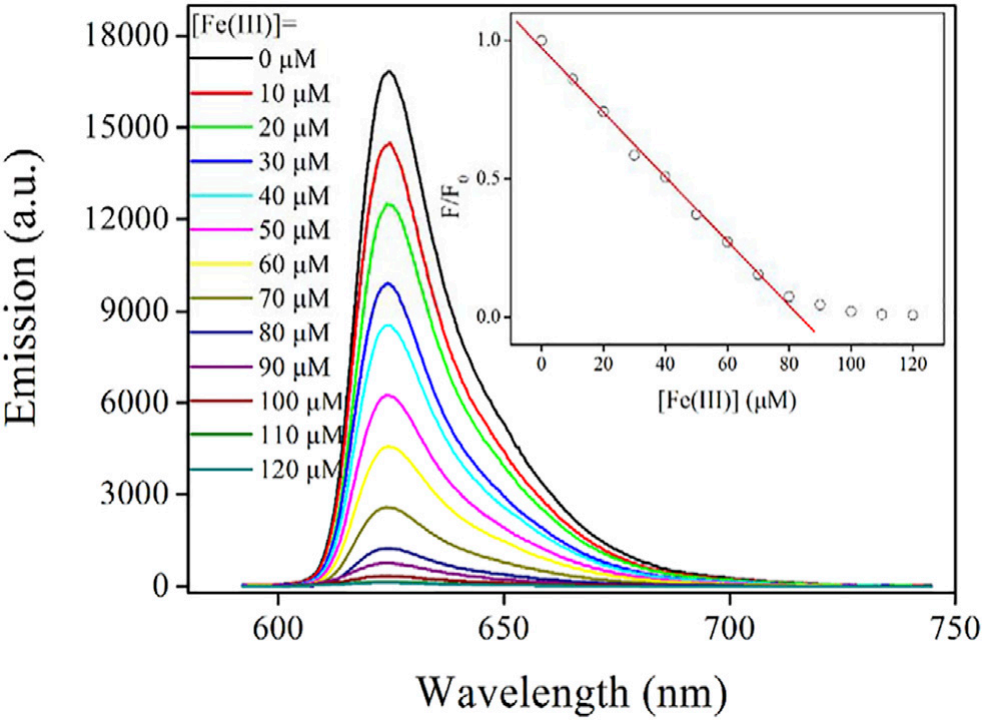
Emission spectra of the AuNCs diluted by pure water (v:v = 1:10), upon increasing Fe(III) concentration from 0 to 120 μM. Inset: Stern–Volmer equation fitted with Formula 4.

As mentioned before, the emission mechanism of the as-synthesized AuNCs is the ligand-to-metal nanoparticle core charge transfer. Here, the ligand (L-Cys) serves as energy-harvesting antenna for emissive center (AuNCs). If the excitation energy of this ligand is quenched by a proper energy acceptor, its energy transfer to emissive center will be ceased as well, leading to the emission quenching of AuNCs. Guided by this hypothesis, a series of metal cations have been used as energy acceptors. It is found that Fe(III) ion quenches AuNC emission efficiently. Figure 8 shows the emission spectra of AuNCs upon increasing Fe(III) concentration from 0 to 120 μM. The emission band peaking at 625 nm is gradually weakened with increasing Fe(III) concentration. At Fe(III) concentration of 120 μM, the remaining emission intensity is only 0.7% of the original emission intensity value. Regardless of this complete emission quenching of AuNCs by Fe(III), no new emission band or shoulder peak is observed. In addition, no emission shift is observed. This fact means that there is no strong interaction between the emissive center of AuNCs and quencher Fe(III). In other words, the observation of this monotonical emission decrease actually indicates a static quenching mechanism through the energy quenching of LMNCT, which will be confirmed later. This hypothesis is consistent with the fact that AuNCs are embedded into, and thus surrounded by the framework formed by L-Cys.

#### Sensing Mechanism: Lifetime Comparison and Emission Recovery by EDTA

**SCHEME 2:**
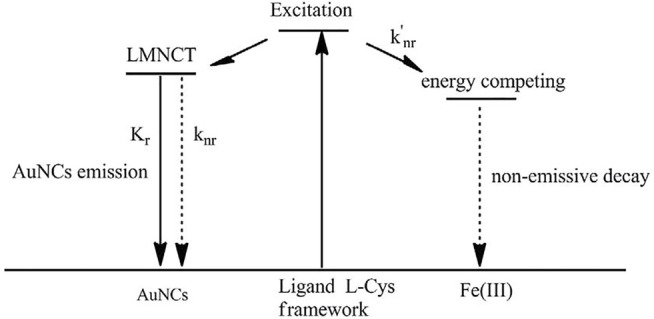
Proposed sensing mechanism of AuNCs toward Fe(III).

To confirm the previous hypothesis about the sensing mechanism (LMNCT static quenching), the emission decay dynamics curve of AuNCs is recorded under Fe(III) concentration of 100 μM and compared with that without Fe(III). It is observed from Figure 7 that the emissive center is greatly quenched by Fe(III), and its lifetime is shortened greatly as well. Corresponding fitting parameters are listed in Table 2. AuNC emission still follows a biexponential decay pattern in the presence of Fe(III), but its decay lifetime is greatly decreased to 99.34 ns. The fast-decay component (τ_2_ = 78.76 ns) is comparable to that without Fe(III) (τ_2_ = 65.89 ns), but the slow-decay component (τ_1_ = 149.34 ns) is much shorter than that without Fe(III) (τ_1_ = 477.67 ns). This fast-decay component has been assigned as the direct transition of AuNCs (sp to sp or d to sp), while the slow-decay component is regarded as the transition of LMNCT. It is observed that the transition of AuNCs is well-preserved during the covering effect of the surrounding L-Cys framework, while the LMNCT transition has been greatly suppressed by Fe(III). Correspondingly, it is found that the K_r_ value of AuNCs in the presence of Fe(III) (3.83 × 10^5^ s^−1^) is also comparable to that without Fe(III) (3.77 × 10^5^ s^−1^), while the calculated K_nr_ value (K_nr_ = k_nr_+k_nr_’) of AuNCs in the presence of Fe(III) (1.06 × 10^7^ s^−1^) is much higher than that without Fe(III) (1.76 × 10^6^ s^−1^). This fact suggests an energy competing procedure (k_nr_’) for the emissive center. It is thus assumed that Fe(III) here serves as an energy acceptor and offers an energy competing path for the emissive AuNCs. With the weakened energy transfer of LMNCT, the emission quenching signal is observed. A schematic presentation for this sensing mechanism is drawn as Scheme 2.

**FIGURE 9:**
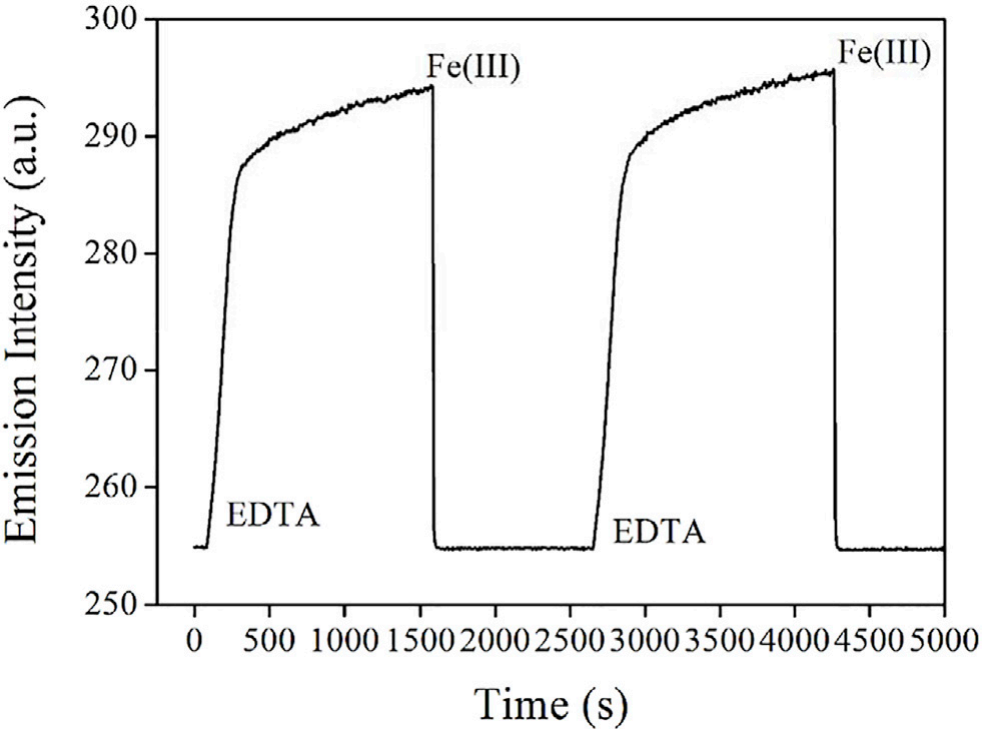
Emission intensity monitoring of the AuNCs diluted by pure water (v:v = 1:10), with Fe(III) and EDTA periodically added.

Guided by this sensing mechanism, the emission quenching of AuNCs is caused by the decreased energy transfer of LMNCT, while the emissive center itself is not quenched or decomposed. If the energy competing procedure for this LMNCT process is stopped, AuNC emission can be recovered. To confirm this hypothesis, AuNCs emission is monitored with Fe(III) and EDTA periodically added. It is observed from Figure 9 that after adding a chelating reagent EDTA, AuNC emission is instantly increased for the first 270 s, then AuNCs emission is gradually increased for the following 1360 s, and finally becomes constant. A similar cycle can be completed when more Fe(III) are added into AuNCs. This result tentatively confirms that the emissive AuNCs are immune to Fe(III) influence. Their emission quenching is simply caused by Fe(III) energy competing for the LMNCT process, which agrees well with the proposed sensing mechanism shown in Scheme 2.

#### Stern–Volmer Equation

With the aforementioned static quenching mechanism confirmed, the emission spectra of AUNCs upon various Fe(III) concentrations can be analyzed using the Stern–Volmer equation. Generally, the intensity form of the Stern–Volmer equation for a sensing probe following a static sensing mechanism is described as Formula 4. Here, F means emission intensity, F_0_ denotes the emission intensity without any quencher, C is a constant, K_sv_ is a Stern–Volmer constant, and [Fe(III)] stands for Fe(III) concentration, respectively.

$$F/F0=C+K_{sv}\left[Fe\left(III\right)\right]$$ (4)

It is observed from Figure 8 that within [Fe(III)] region of 0–80 μM, F/F_0_ follows a linear curve. With these emission spectra on hand, this working equation is fitted as F/F_0_ = 0.9730–0.0116*[Fe(III)], *R* ^2^ = 0.9978. The corresponding working region is 0–80 μM. At Fe(III) concentration higher than 80 μM, F/F_0_ is slightly decreased and finally becomes constant. This fact means that AuNCs have reached their ceiling sensing capacity. Following a literature method (3σ/N), the limit of detection (LOD) value is determined as 3.9 μM (corresponding to 217.6 ppb). This value is found comparable or even lower than values of similar sensing systems for Fe(III) optical detection given in the literature (Xie et al., 2007; Zhang et al., 2014).

#### Selectivity

**FIGURE 10:**
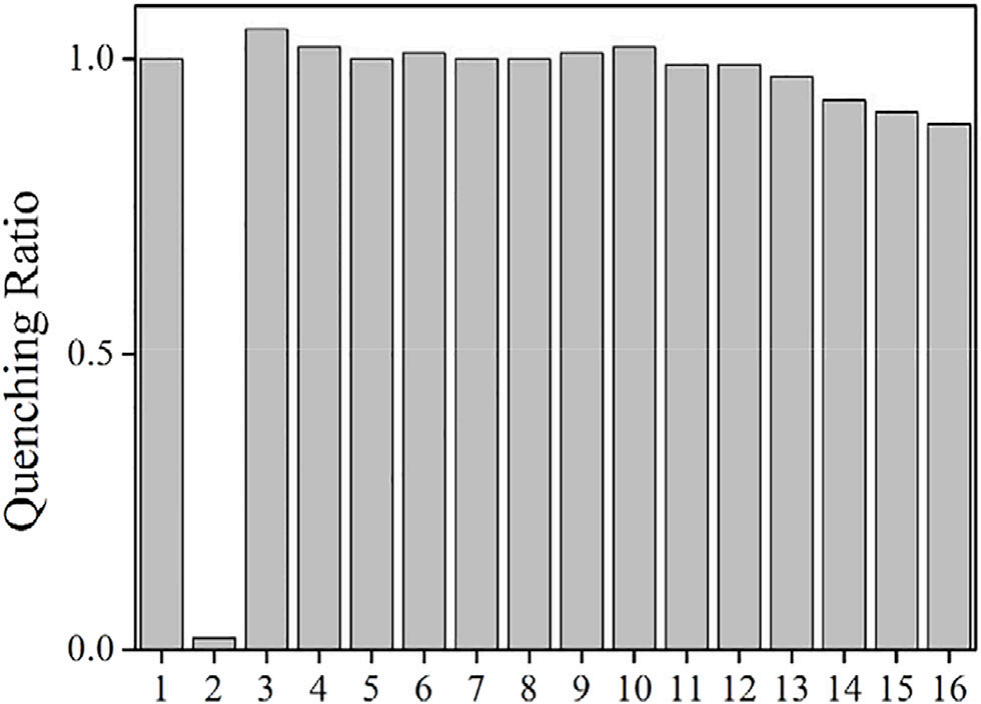
Emission intensity quenching ratio of the AuNCs diluted by pure water (v:v = 1:10), in the presence of metal cations and anions (100 μM). 1 = blank, 2 = Fe^3+^, 3 = AcO^−^, 4 = PO_4_^−^, 5 = SO_4_^−^, 6 = NO_3_^−^, 7 = Na^+^, 8 = Mg^2+^, 9 = Ca^2+^, 10 = Zn^2+^, 11 = Cd^2+^, 12 = Co^2+^, 13 = Al^3+^, 14 = Cu^2+^, 15 = Fe^2+^, and 16 = Hg^+^.

The as-synthesized AuNCs have shown promising sensing performance toward Fe(III), and there is a problem to be solved though. For most emission quenching sensing systems, their selectivity always faces a challenge since their emission may be quenched by emission killers or energy competing systems, leading to fake signals and thus unreliable result. In this case, the interfering influence of other metal cations and anions on AuNCs is evaluated by adding them into AuNCs, including AcO^−^, PO_4_^−^, SO_4_^−^, NO_3_^−^, Na^+^, Mg^2+^, Ca^2+^, Zn^2+^, Cd^2+^, Co^2+^, Al^3+^, Cu^2+^, Fe^2+^, and Hg^+^. It is observed from Figure 10 that AuNCs emission is immune to most anions, including AcO^−^, PO_4_^−^, SO_4_^−^, and NO_3_^−^. The presence of AcO^−^ even slightly enhances AuNCs emission. As mentioned before, the zeta potential of AuNCs is measured as −4.0 ± 0.5 mV. These anions may stabilize AuNCs, leading to the stable emission of AuNCs. On the other hand, most metal cations have quenching effect on AuNCs, especially for Al^3+^, Cu^2+^, Fe^2+^, and Hg^+^. This is because there is strong electrostatic interaction between these metal cations and AuNCs, leading to possible energy transfer from AuNCs–LCys framework to these metal cations and thus emission quenching of AuNCs. On the other hand, their quenching effect on AuNCs is more ineffective than the Fe^3+^ quenching effect (0.89 vs 0.02). This is because Fe^3+^ has strong electrostatic interaction with AuNCs and thus quenches their emission efficiently. The other metal cations and anions fail to have such strong electrostatic attraction with AuNCs, leading to a weak interaction between them. No effective energy transfer can be realized between AuNCs–LCys framework and interfering ions. A good sensing selectivity of AuNCs toward Fe^3+^ is thus confirmed.

For the convenience of comparison, some key sensing parameters of the as-synthesized AuNCs are compared with those of sensing systems given in the literature, as shown in Table 3. It is observed that the sensing performance of AuNCs is comparable to that of optical sensing systems based on organic dyes. The major advantage of AuNCs is the simple and fast synthetic procedure and promising sensing performance. Regardless of the emission turn off sensing behavior of AuNCs, a good sensing selectivity is still observed.

**TABLE 3:** Some key sensing parameters of AuNCs and literature sensing systems.

System	Analyte	Sensitivity region	Linearity	Selectivity	Ref
AuNCs@BSA	Ag^+^	0.58–1.30	no	N/A	13
HAS-AuNCs	NO_x_	0.4–1.8	yes	good	14
BSA-SuNCs	Cu^2+^	∼2.4	linear-liked	limited	15
DHLA-AuNCs	Hg^2+^	20–100	yes	limited	19
AuNCs	Fe^3+^	0.02–1.00	yes	good	this work

## Conclusion

To sum up, AuNCs with a mean diameter of ∼2 nm were prepared successfully by using a simple method of microwave-assisted reaction. We used L-cysteine as both a reducing agent and stabilizer. These AuNCs were characterized with methods of TEM, XPS, DLS, and IR. Their UV-vis absorption, emission, quantum yield, and lifetime were discussed as well. Bright red emission was originated from ligand-to-metal nanoparticle core charge transfer (LMNCT), with a quantum yield of 17.6% and an average decay lifetime of 466.99 ns. It was then found that such LMNCT process can be quenched by Fe(III) cation. An effective working region of 0–80 μM with an LOD of 3.9 μM was finally observed. Their quenching mechanism was revealed as Fe(III) energy competing for the LMNCT process. The novelty and advancement of this work is the simple synthesis and impressive sensing performance, including wide working region, good linearity, and selectivity. For further research, efforts should be devoted to the improvement of sensitivity. Selectivity is yet to be satisfied and can be improved as well.

## Data Availability

The datasets presented in this study can be found in online repositories. The names of the repository/repositories and accession number(s) can be found in the article/Supplementary Material.
